# An intertextual reading of the novel *Defend the Name*

**DOI:** 10.1371/journal.pone.0304177

**Published:** 2024-06-17

**Authors:** Ayenew Guadu

**Affiliations:** Department of English Language and Literature, Faculty of Humanities, Bahir Dar University, Bahir Dar, Ethiopia; Bahir Dar University, ETHIOPIA

## Abstract

The general objective of this intertextual analysis’s was to explore Wolde’s novel *Defend the Name* (1969) with the view to identify and interpret the several thematic and stylistic intertexts that are woven throughout the narrative. Based on available research, there is a scarcity of critical studies that have utilized the theory of intertextuality for the analysis and interpretation of Ethiopian prose fiction in English, particularly within the novel genre. The current study was aimed to partially fill in this critical gap. In doing so, the theory of intertextuality is employed as theoretical-analytical framework of the study. The findings of this intertextual analysis concentrated on the thematic and stylistic intertexts that were woven throughout the plot of the book *Defend the Name*. These intertexts included biblical allusions, colonial literary devices, contemporary theoretical and ideological works, and cultural and historical discourses that the book intertextually engages with in addition to other literary and nonliterary works. This study provides insightful information about the thematic diversity of Defend the Name and its involvement with multiple intertexts through its intertextual analysis. It enhances the reader’s comprehension of the story, characters, and larger sociopolitical situations that the novel addresses, demonstrating the author’s skill in fusing together a variety of literary, scriptural, ideological, and cultural aspects.

## 1. Introduction

Literature, influenced by postmodernist philosophical ideas, has undergone a significant reevaluation of its theoretical, critical, and functional concepts. One prominent postmodernist literary theory is intertextuality, which has garnered increasing interest and enthusiasm among writers, critics, theorists, lecturers, and students of literature worldwide [1, 2]. Intertextuality explores the thematic and stylistic connections between literary texts and their rhetorical implications in determining each text’s success. According to Lourdes López-Ropero, critics have highlighted literature’s ability to rejuvenate and revitalize literary tradition through retelling [3, 4].

Postmodernist critics and theorists assert that intertextual links between literary texts have become integral, to the point where it is difficult to imagine a literary text without such connections [5–7]. Coined by Julia Kristeva’s essay, the term *intertextuality*, in literary theory and criticism, refers to the presence of a prior text’s subject matter, themes, ideology, and echoes of its stylistic and formal elements in a subsequent text. Consequently, the later text contains explicit or implicit references to these features from other prior texts. From a historical perspective, intertextuality implies that literary texts are always constructed within a specific context or literary tradition [7].

Intertextuality encompasses the way in which texts of various forms (oral, visual, literary, virtual) reference other texts that have contributed to their creation and meaning [8, 9]. There are specific aspects of intertextual connections between texts that literary critics, teachers, and advanced students can explore. Before delving into these categories, it is useful to consider Cuddon’s distinction between “form” and “substance”. *Form* refers to the shape, structure, style, and genre of a literary work, while *substance* pertains to its content or what it is about [10].

To simplify the analysis of intertextuality, these aspects can be grouped into two broad categories: thematic intertextuality and stylistic/formal intertextuality. Thematic intertextuality involves the shared substance or content between two or more texts. It examines the thematic elements, such as references to specific facts or events, narratives, or works within a particular literary tradition [10–12].

Thematic and stylistic intertextuality can be seen between oral and written/visual mediums, as well as between visual and oral/written modes of expression [13–15]. Oral storytelling traditions influence the stylistic choices of written works, as authors incorporate the rhythmic patterns and vivid imagery of oral narratives into their writing. This exchange between oral and written traditions enhances the immersive experience for the reader [15]. Similarly, visual art inspires writers and poets, who incorporate visual imagery into their literary works, conveying complex themes and ideas through words. Writers, in turn, can influence visual artists by providing narratives or poems that complement visual elements [16]. This interplay between mediums enriches the artistic landscape, fostering a dialogue that transcends boundaries and encourages diverse sources of inspiration, resulting in works that resonate on multiple levels.

On the other hand, stylistic/formal intertextuality focuses on the textual or structural elements shared by the texts under examination. It involves analyzing aspects of style, quotations, pastiche, allusions, irony, satire, parody, tone, imagery, and references to the form, aesthetics, style, and genre of a particular period [10, 12].

In conclusion, intertextuality has become a vital area of study in literature, driven by postmodernist philosophical premises [1]. It encompasses both thematic and stylistic/formal connections between texts, emphasizing their impact on the success and meaning of each work. By exploring these aspects, literary critics, teachers, and advanced students can gain a deeper understanding of the intricate relationships between literary texts [2, 4, 17].

## 2. Objectives of the study

The general objective of this intertextual analysis was to explore Wolde’s novel *Defend the Name* [18] with the view to identify and interpret the several thematic and stylistic intertexts that are woven throughout the narrative. The specific objectives of study are to:

- Examine the biblical intertexts in *Defend the Name* and assess their significance in relation to the themes and narrative structure of the work;
- Analyze the colonial literary tropes used in the work in the novel under analysis and consider how they contribute to its thematic development.
- To investigate the allusions to contemporary theoretical and ideological works in the novel and assess how they impact the story and dialogue of the novel.

## 3. Scope of the study

The scope of this study is focused on conducting an intertextual reading of the novel Defend the Name by examining its intertextual references in four subtopics. Firstly, it explores colonial literary tropes as thematic intertexts, analyzing how the novel engages with and challenges conventional colonial literary conventions. Secondly, it examines biblical intertexts to understand their significance within the narrative. Thirdly, it investigates the novel’s references to modern ideological and theoretical texts and their influence on themes, character development, and social commentary. Lastly, it explores the role of cultural and historical discourses as sources of intertextuality, considering their impact on the narrative. Through these approaches, the study aims to uncover the intertextual dimensions of Defend the Name and their thematic implications.

## 4. Text selection

The novel *Defend the Name* was selected as the subject of this intertextual reading due to its rich intertextual elements and its potential to contribute to the understanding of intertextual practices in Anglophone African literature. The presence of intertextual references and connections within the text provides an opportunity for in-depth analysis and exploration. Furthermore, the novel’s relevance to broader discussions on intertextuality played a significant role in its selection.

## 5. Literature review

### 5.1. Postmodernism and intertextuality

"[A] Postmodern age" [19] is a term used by scholars to label the contemporary historical period we are living in. Jean-François Lyotard is credited with inaugurating the postmodern era in the last quarter of the 20th century with his book *The Postmodern Condition*: *A Report on Knowledge* [20]. However, pinpointing the exact starting point of postmodernism in history remains challenging for scholars. Postmodernism, as a philosophical notion, encompasses a range of transformative intellectual movements [21] that share a common purpose in critiquing the core values and belief systems of modernist approaches, such as rationalism, objectivity, and the idea of scientific and social progress [17, 21].

In literary studies, the impact of postmodernism is evident in the rejection of privileged positions and values traditionally attributed to Western grand narratives and canonical literary texts. Starting from the mid-1960s, traditional and totalizing notions of the originality of literary texts and the values of grand narratives were unsettled and undermined [22]. Intertextual literary theory is strongly associated with both postmodernism and poststructuralism (or deconstruction). Poststructuralism is considered an aspect of postmodernism [19, p. 181]. Similarly, poststructuralism is a broader philosophical and literary critical notion that encompasses the theory of intertextuality. Intertextuality, rooted in the works of French poststructuralist literary theorists such as Julia Kristeva, Roland Barthes, and Jacques Derrida, emerged as a poststructuralist literary critical term and found major contributions in the elitist and avant-garde journal *Tel Quel*, published in Paris in the mid-1960s [19, 21, 22].

### 5.2. Theory of intertextuality

The term "intertextuality" emerged during the 1960s and is often associated with the postmodern age [19]. However, intertextuality as a practice in literary production is not exclusive to the twentieth century but has existed throughout recorded human society [18]. The study of intertextuality can be found in theories and discussions about texts throughout history. Therefore, many books on intertextuality acknowledge the contributions of early writers to provide a historical perspective on intertextuality theories [17, 18].

While references to classical, medieval, and Renaissance writers can be found in authoritative books on intertextuality, most authors focus primarily on twentieth-century and present-day theorists such as Mikhail Bakhtin, Julia Kristeva, Roland Barthes, Jacques Derrida, Gérard Genette, Michael Riffaterre, Harold Bloom, and Jonathan Culler [19, 23, 24].

By implication, the twentieth century has witnessed a proliferation of diverse notions and theories of intertextuality, leading to the use of the plural form "theories" or "intertextualities" to describe the phenomenon [17]. Furthermore, scholars have attempted to categorize later theories of intertextuality into different lines of development. For instance [19, 23], both identify two main categories: (1) the "poststructuralist–deconstructive" strand of intertextual theory, exemplified by Roland Barthes [23] and (2) the more narrowly defined "structuralist approaches" and "poetics" of intertextual analysis, illustrated by Genette and Riffaterre [19] Due to lack of space, the brief review in the upcoming paragraphs focuses on only four of the aforesaid influential twentieth-century theorists: Mikhail Bakhtin, Julia Kristeva, Roland Barthes, and Gérard Genette. Out of these four, Bakhtin and Kristeva are regarded as the originators of the literary theory of intertextuality, while Barthes and Genette represent later developments in intertextual theories [19, 23].

To begin with, Mikhail Bakhtin’s theory of intertextuality centers around key concepts such as polyphony, heteroglossia, and the dialogic novel [25]. Bakhtin argues that a narrative contains multiple voices and worldviews in constant conflict, with each voice influenced by and responding to others. These voices and discourses are intertextually connected, shaping the meanings within the text. Bakhtin draws upon the subversive traditions of the carnivalesque to exemplify the struggle for meaning and its impact on language and communication [25].

Julia Kristeva’s theory of intertextuality emphasizes the idea that a literary text is essentially intertextual, functioning as a "mosaic" of direct and indirect references to other texts, genres, and discourses. The author draws from prior texts during the process of composing a text, rearranging elements with socially pre-existing meanings. Kristeva’s semiotic approach highlights the transformative nature of intertextuality [26, 27].

Roland Barthes’s theory of intertextuality focuses on the "plurality of the text" [28, 29]. He argues that a text achieves a multiplicity of meanings that are irreducible and interconnected. Barthes emphasizes the intertextuality inherent in the "textual space," where a text generates multiple meanings when read in relation to other texts, past and present. Barthes and Kristeva both highlight how a text absorbs and transforms elements from other sources, emphasizing the inherent multiplicity within poetic language [28, 29].

Gérard Genette’s concept of "transtextuality" provides a comprehensive framework for analyzing intertextual connections in texts. Genette’s work on intertextuality, particularly his *Palimpsests* [29]. identifies and defines various intertextual relationships such as parody, allusion, and pastiche. His framework enables the analysis of how a text engages with other texts, whether through explicit references, subtle allusions, or the creative reworking of literary conventions [30–32].

In summary, the theoretical framework of intertextuality encompasses the contributions of influential theorists such as Bakhtin, Kristeva, Barthes, and Genette. These theorists explore different aspects of intertextuality, including polyphony, dialogism, the transformative nature of language and discourse, and various intertextual relationships [11]. Understanding intertextuality enhances our understanding of how texts engage with prior texts and the broader cultural and literary landscape.

### 5.3. Theoretical framework: Applicability of theories of Bakhtin, Kristeva, Barthes, and Genette to the reading of novel *Defend the Name*

Intertextual theories by Bakhtin, Kristeva, Barthes, and Genette are readily applicable to the analysis of the thematic and stylistic intertexts in the novel *Defend the Name* as described herewith. Bakhtin’s concepts of polyphony and dialogism can be fruitfully applied to the intertextual analysis of *Defend the Name*, for the story involves conflicting perspectives on the impact of colonialism. For example, the clash of voices—colonizers versus colonized—creates layers of meaning and allows us to explore the complexities of colonial narratives. Likewise, Julia Kristeva views texts as mosaics composed of direct and indirect references to other texts whereby authors draw from prior works, rearranging elements with pre-existing meanings. In the novel *Defend the Name*, her concepts can be readily applied to the analysis of biblical intertexts.

Roland Barthes emphasized the plurality of the text by which he means that meaning doesn’t reside solely within the text; it emerges through the reader’s interaction with a complex network of invoked texts. His concepts are applied to interpreting to novel’s references to modern ideological and theoretical texts. By examining how these references generate multiple meanings in relation to other texts, we gain insights into the novel’s engagement with contemporary intellectual discourses. Similarly, Gérard Genette’s concept of *transtextuality* implies the various intertextual relationships between two or more texts. For example, his concept helps to analyze the novel’s engagement with cultural and historical discourses—such as the “Self-Made Man,” colonialism, and missionary narratives—that we uncover in narrative of the novel under study.

The intertextual theories of Bakhtin, Kristeva, Barthes, and Genette offer valuable frameworks for comprehending the role of intertexts in *Defend the Name*, shaping its overall meaning, themes, and stylistic choices. By employing these theories, we can explore the intricate interplay between texts, voices, and discourses within the novel, enhancing our understanding of its literary and cultural significance. Bakhtin highlights the dialogic nature of intertextuality, Kristeva unravels layers of meaning through signification and genre intermingling, Barthes considers external influences on authorial intent and reader interpretation, and Genette’s transtextuality framework examines how *Defend the Name* interacts with other texts, shaping its literary identity. By eclectically employing these theorists, we gain a comprehensive understanding of intertextual dynamics in Defend the Name.

## 6. Synopsis of the novel *Defend the Name*

Wolde Haile’s novel, *Defend the Name* [33] is set in the imaginary city of Babilé, named after its ruler, Lady Babilé. The novel tells the tragic story of Babilé, a nation and its leader, who are disgraced, oppressed, and exploited by Jonathan, a self-appointed missionary. Initially posing as a savior sent by God, Jonathan gains the trust of Lady Babilé and the community. However, his true nature is gradually revealed as he exploits his position of power.

Jonathan brings a group of white soldiers to Babilé, asserting his control over them. He holds a gathering where he boasts about his tyrannical leadership and arrogantly renames the city as "The City of Jonathan." The black natives suffer under Jonathan’s colonialist megalomania, enduring exploitation, alienation, and his brutal and cruel acts. The oppressed natives attempt a revolt and an unsuccessful assassination of Jonathan, but their resistance is crushed. Tragically, the novel concludes with Lady Babilé being executed, intended to suppress further anti-colonial protest and resistance.

Overall, this story resonates intertextually with Frantz Fanon’s *The Wretched of the Earth* [34], which vividly depicts the impact of colonialism on the black race, highlighting the suffering inflicted by Western colonizers.

## 7. An intertextual reading of the novel *Defend the Name*

### 7.1. Colonial literary tropes as thematic intertexts in *Defend the Name*

#### 7.1.1. The trope of “*Things Fall Apart*”

The arrival of colonialism in Africa inevitably led to the disruption of indigenous socio-cultural identities and political systems within African societies. Consequently, numerous colonial and postcolonial Anglophone African novels have focused on revealing the devastating effects of colonialism on the lives of the colonized. One such novel is *Things Fall Apart* [35] by Chinua Achebe, which aptly captures the socio-politico-cultural disruptions and crimes against African people precipitated by the arrival of the white man in Africa.

Achebe perceives his writing career primarily as a political mission, aiming to challenge the misinformed, dangerous, and baseless view of Africa held by the West as "the heart of darkness" populated by savages lacking intelligible socio-political systems [36, p. 53]. Similarly, Ngugi, who believes that "the black man did not really exist, had slept in the dark continent," views the task of the African writer as restoring the African character to his history and reestablishing the broken dialogue with the gods of his people [36, p. 58].

Thus, *Things Fall Apart* can be considered an intertextual work that falls within the lineage of preceding Anglophone African novels dedicated to challenging Western stereotypes and perceptions of Africa as "the dark continent" inhabited by savages. [36], supports this classification by asserting that unlike the Western portrayal of Africa as "the other world," a place of negations peopled by savages, *Things Fall Apart* presents a people with a well-defined culture and an articulate political system.He groups the novel among works that aimed to rehabilitate the identity and history of the African character, assert the validity of African cultures, and expose the violence inflicted by colonial domination on African societies [quoted in 36, p. 53].

Wolde’s *Defend the Name* also intertextually partakes of the same anti-colonial literary project set by *Things Fall Apart* and other works, as analyzed hereafter. To begin with, *Defend the Name* is peppered with passages that describe the disruptions of the indigenous socio-cultural values and norms, such as the solidarity of the community, as well as the peaceful relationships and threads that used to hold the family intact before the Advent of the white colonizers. Most importantly, these situations can be illustrated by an extract of the conversation held among the elders of the community of Babilé:

“Surely, we want to live in the old fashion,” augmented still another.

“Yes really, before the advent of colonialism, we lived like members of one family. But now there is hatred amongst each other. There is hatred even between us and those who are akin to us. My children do not go along with me, which is also the case with you people.”

“We should restore and defend old ways of living. We should restore and defend the name Babilé.

“We must find *the hidden devil* that is here and that makes us quarrel and that disturbs our peace. We must find *this mischievous devil and we must kill it*. *We must kill it*, I repeat it.”

“Yes! Yes! Yes! Yes!! Yes!!!” [*emphasis added*] [33, p. 76]

This conversation among the elders of the community, according to the narrator, culminated in “a great march to triumph over *an invisible devil*.”[*emphasis mine*]. Then, as the reader can see from both quotations here, there are three references to the *devil* (which include “the hidden devil,” “this mischievous devil” and “an invisible devil”), of which the first two are made by the elders and the last one by the narrator of the story. Bearing the subject of the above quotation from *Defend the Name* (by Wolde) in mind, the reader can compare and contrast it with the following quotation from *Things Fall Apart* (by Achebe):

“This is a great gathering. No clan can boast of greater numbers or greater valor. But are we all here? I ask you: Are all the sons of Umuofia with us here?” a deep murmur swept through the crowd.

“They are not,” he said. “They have broken the clan and gone their several ways. We who are here this morning have remained true to our fathers, but our brothers have deserted us and joined a stranger to soil their fatherland. If we fight the stranger we shall hit our brothers and perhaps shed the blood of a clansman. But we must do it. Our fathers never dreamt of such a thing, they never killed their brothers. But a white man never came to them. So we must do what our fathers would never have done. Eneke the bird was asked why he was always on the wing and he replied: ‛Men have learnt to shoot without missing their mark and I have learnt to fly without perching on a twig.’ *We must root out this evil*. And if our brothers take *the side of evil we must root them out too*. And we must do it now. *We must bale this water* now that it is only ankle-deep…” [*emphasis added*]. [35, pp. 167–68]

Intertextually, the subject of discussion at the gathering of the clan of Umuofia in *Things Fall Apart* appears to be very similar to that of the community elders in *Defend the Name*. Secondly, in the passage quoted from *Things Fall Apart*, there are two references to *evil* (“this evil” and “evil”) that befell the clan of Umuofia, and because of which, the elder requests: “We must root out this evil. And if our brothers take the side of evil, we must root them out too.” Then, these references to *the evil* in Achebe’s novel might be intertextually evoked in *Defend the Name* by those references to *the* d*evil* (“the hidden devil,” “this mischievous devil” and “an invisible devil”), as shown in the passage quoted above, in which the community elder requests: “We must find *the hidden devil* that is here and that makes us quarrel and that disturbs our peace. We must find *this mischievous devil and we must kill it*. *We must kill it*, I repeat it.” [*emphasis added*].

After all, the analysis of this thematic intertextual relationship between Wolde Haile’s *Defend the Name* and Chinua Achebe’s *Things Fall Apart* reveals their striking similarities and underscores the significance of examining their intertextual connections. Both novels share a common objective of challenging Western stereotypes of Africa and portraying the destructive consequences of colonialism on indigenous societies. By situating itself within a literary lineage of resistance and cultural reclamation, *Defend the Name* aligns with the broader movement of African writers seeking to reclaim African identity, challenge colonial narratives, and validate African cultures. This analysis emphasizes the interconnectedness of African literature and its collective effort to address the complex legacies of colonialism.

#### 7.1.2. The trope of “Living a dog’s life”

The author of *Defend the Name* has metaphorically compared the life of the colonized natives with the life of dogs by the help of which he is able to depict the vivid images of alienation, dehumanization and unfair treatment to which the colonized people were subjected. In so doing, the novelist first showed how the natives were alienated from their own food resources, which can be demonstrated best by the supper meal described in the third chapter of the novel, as explained below.

This meal was served at lady Babilé’s house on the day Jonathan (who “did not want to be honest and just” until then) made the natives to listen to (and recite) a poem he wrote. The first important thing to be noticed in this description of the meal is that the author’s employment of a sharp contrast in the quality and quantity of food served to Jonathan and to the natives serves as a narrative strategy for portraying the latter’s alienation from their own food resources and from the fruits of their work. It is also important to observe in the same description that the native Babilées, having been subjected to colonial alienation and deprivation, were portrayed to survive by scavenging on leftovers and meatless bone thrown out by the colonialist:

All the Babilées displayed a facial description of appreciation. Supper had to be served; for, this recital was fatiguing. Tolulu got the meal ready under the order of Jonathan. His meal was completely different from that of the Babilées. His was nice and tasty, sweet and delicious, varied in sorts; whereas theirs was unattractive and unbalanced. His food was honorably placed on the table whereas theirs on the floor. He cleaned off all the meat from a bone. He ate the meat and threw the bone. And since the Babilées were all eager for such an occasion, they rushed to it madly. Each wanted to have it for himself—egocentricity had highly developed. There was a battle of looks among them. Even though “Tout comprendre, c’est tout pardonner,” which is the devil’s sentimentality sounded reasonable, Nathnael’s feelings were a bit harrowed and he became very inquisitive.

“Mamma,” he said one day.

“Yes mine,” responded Babilé.

“*Why is it that we have to fight for a bone which has no meat on it*? *In the past we used to get meat freely and in plenties*.” [*emphasis added*] [33. P. 15]

This brings the reader to an important (linguistic and thematic) intertextual feature indicated in title of the present subsection; namely, an idiomatic expression *to live/lead a dog’s life*, which appears three times in the novel under analysis. To begin with, as defined in the *OED*, the idiomatic phrase “a dog’s life” means “an unhappy life, full of problems or unfair treatment.” Most importantly, in his *A Dictionary of Literary Symbols* (2007), Michael Ferber writes: “To go to the dogs, to die like a dog, to *lead a dog’s life*—these and similar phrases are common expressions of the *miserable status of dogs*” [*emphasis added*.] [37, p. 60]. In this respect, when examining Defend the Name, three references to the phrase "to live/lead" a dog’s life are present. The first two references are made by Lady Babilé herself, while the third is made by one of the college students, as explained in their respective contexts.

Firstly, upon the hypocrite Jonathan’s proclamation of Christianity to the black natives therein, the queen regnant lady Babilé expresses her willingness to embrace the faith and thereby “to live a dog’s life” rather than to live under the dominion/powers of vice/evil and ignorance:

“I shall obey the orders of Father Jonathan. I shall wash his feet and clothing; I shall serve him to the best of my ability; I shall try to make him always happy; as for me, I shall bear any burden and I shall *live a dog’s life*. Long live our teacher who has liberated us from the dominion of vice, who has emancipated us from the powers of evil and ignorance.” [*emphasis mine*.] [33, p. 16]

Ironically, by the time Jonathan started to exercise his colonial tyranny, the now disgraced lady Babilé was heard condemning the miserable life which they were reduced to and which was comparable to “a dog’s life” (and/or a slave’s life). She, then, began to encourage her people to struggle for their freedom, saying: “Let’s rise from our graves; let us put an end to agonizing and torturing miseries; let’s not live a dog’s life. Let us live a noble life or die a noble death. Let us not live a slave’s life and die a slave’s death. I am wholly and entirely ready to challenge death as Wellington did in Waterloo” [33, p. 26].

The last (or third) reference to “a dog’s life” the colonized natives were doomed to lead was made by one of college students (who were not only “imbedded in Marxist aspirations” but also the progressive ones and the most agitators, as remarked by the narrator at the first paragraph of the sixth chapter). It is in the middle of a conversation between two students that one of them requests his friend, “Cigarettes, you know I have not smoked for the last three days. And I am in the habit. Can you buy one for me?” Much to the chagrin of the requester, his friend replies:

“Man, if I had cents, *I would have eaten my lunch*.”

“It is terrible! Thus do you call this life?”

“Yes, but it is *a dog’s life*.” [*emphasis mine*.] [33, p. 85]

Now the reader is in a position to comment on the significance of the idiomatic phrase “a dog’s life,” as it has been repeatedly encountered in the narrative of *Defend the Name*. That is, by recycling this idiomatic phrase with a set meaning, Wolde has been able to paint the vivid pictures of the marginalized existence and disgraceful status of the colonized (or, to borrow Franz Fanon’s words, “the wretched of the earth”).

In sum, the analysis of "living a dog’s life" in Defend the Name reveals its intertextual significance in colonial literature, depicting the marginalized existence and oppressive treatment of colonized communities. This dialogue with works like Things Fall Apart highlights African writers’ collective effort to challenge colonial narratives, reclaim identity, and expose the realities of colonization. Through intertextual connections, readers gain a deeper understanding of themes and socio-political implications, contributing to a comprehensive understanding of colonialism’s impact on marginalized communities.

#### 7.1.3. The tope of colonial viciousness and atrocity

It is undeniable that works of literature inevitably grapple with various aspects of human nature, which is why literature falls under the purview of the Humanities. Consequently, it becomes imperative to explore significant facets of human nature that frequently recur in literary works, particularly novels, in order to discern the intertextual significance of these themes. Among the most prominent themes pertaining to human nature that consistently traverse diverse works of literature are vice/evilness, death, and love. In this specific intertextual analysis of Wolde’s *Defend the Name*, the focus is on the literary trope of colonial viciousness and atrocity as an exploration of vice within the colonial context.

To begin with, the human proclivity to be evil and to do evil things, to begin with, has been reflected by the character lady Babilé in *Defend the Name*, as would be briefly described. It would be recalled that the tragic heroine lady Babilé, because she had been innocent and guileless, was manipulated and subjugated by the hypocrite Jonathan who gradually turns out to be tyrannical colonial master. Then, following the full realization of her self-inflicted disgrace and tragedy, lady Babilé begins to reflect on human proclivity to be evil and to do evil things. That is, one day lady Babilé was having a fireside chat with her son Nathael and her old maidservant Tolulu and, in the meantime, (“boiling” with “a burning fire” within her), lady Babilé stormed angrily, “Human beings are always wolves dressing in sheep’s clothing. I hate them”. Upon this, the bewildered Nathnael asked, “Mamma, you hate human beings?” but her explanatory response reads: “I hate no human beings. But I hate injustice that dwells in human beings. And since injustice is inseparable from the human being who possesses it, I hate him too” (and it should be clear that by “him” here lady Babilé was referring to Jonathan, as the reader can confirm it by reading the next conversational turns between the son-mother dyad [33, pp. 22–23].

From the perspective of intertextuality, lady Babilé’s statement that “Human beings are always wolves dressed in sheep’s clothing” is evocative of what is referred to, in Christianity, as the “original sin” and, as defined by the *OED*, the “original sin” means “the tendency to be evil that is believed to be present in everyone from birth.” Put simply, this dictionary meaning of the “original sin” implies the belief that, naturally “from birth,” every human individual (“everyone”) is essentially constituted of (and harbors) the potential to be evil and to do evil things. This original sin, in other words, is usually believed to have been rooted in (or/and caused by) “the fall of the human race and the cosmos from original innocence” as explained in the passage quoted here:

The problem of evil and the suffering that it causes are likewise major themes of the Bible. The authentic note of human suffering is pervasive. Some of this suffering is simply the result of the fall of the human race and the cosmos from original innocence. Some of it is the result of self-destructive evil that individuals bring on themselves; some of it is inflicted by other people and even by groups or nations. [38, p. 345]

On the other hand, as the readers of the New Testament recall, Jesus Christ spoke thus: “Beware of false prophets, which come to you in sheep’s clothing, but inwardly they are ravening wolves” (Mt. 7:15). Here one can see that, according to the Judo-Christian tradition, *wolf* and *sheep* are metaphors for opposite types of human behaviour, where the *wolf* stands for an opportunistic, shrewish, cunning and ruthless inner nature of humanity, while as a victim the *sheep* represents naivety, innocence, guilelessness, trustfulness and vulnerability. In this respect, the reader can say that such metaphorical representations of inner human behaviour have been intertextually drawn by lady Babilé in her statement “Human beings are always wolves dressed in sheep’s clothing”. Nevertheless, by using a non-qualifying “always” to equate “human beings” with “wolves,” lady Babilé has been unreasonably categorical, the cause of which, in fact, might be because she was annoyed to have discovered that Jonathan was one of the “false prophets” or/and “wolves dressed in sheep’s clothing,” as Jesus Christ calls them. This was why, later on in the story, lady Babilé tells her son Nathnael about the sadistic behaviour of Jonathan and his fellowmen: “They are different beings who extract pleasure out of cruelty” [33, p. 54].

The analysis of the trope of colonial viciousness and atrocity in *Defend the Name* holds significant intertextual significance within the broader context of colonial literature. This analysis resonates with other works that explore the theme of oppression and the inherent evilness of the colonizers. It draws intertextual connections with religious references, such as the concept of "original sin" and Jesus Christ’s warning about "wolves in sheep’s clothing," highlighting the recurring motif of human nature’s potential for evil. By examining these intertextual references, readers gain a deeper understanding of the pervasive nature of colonial violence and the dehumanizing effects it has on both the colonized and the colonizers. This intertextual analysis contributes to a broader discourse on the consequences of colonialism and the exploration of human vices within the colonial context.

### 7.2. Biblical Intertexts in *Defend the Name*

#### 7.2.1. References to both the name and death of Jonathan

Reading the name *Jonathan* being given to a character in fiction (like in *Defend the Name* by Wolde) might probably evoke the Biblical hero of same name whose story is told in the Old Testament. That is, according to the Books of Samuel, the warrior Jonathan is the eldest son of king Saul of Israel and close friend of David, who finally was killed in battle against the Philistines (1 Samuel 13–2 Samuel 21).

What is of great importance for the present purpose is that the Biblical Jonathan was a brave warrior who, having fought heroically, “smote the garrison of the Philistines that was in Geba” so as to help his father free Israel from them. This heroic feat of Jonathan in the Bible seems to have an intertextual echo in *Defend the Name* as described herewith. That is, contemplating what he would do if he were to be faced with enemies in Babilé, the fictional character Jonathan thinks of this: “Jonathan is unbeatable. Let my enemies be careful before it is too late. If there are any, let them be prepared to leave my regime or to entertain a good deal of punishments. For me this will not at all be a painstaking task. Bullets to the culprits, death to the offensive!” [33, p. 5].

In addition, in the First Book of Samuel, the reader finds one intertextually significant question, concerning the death of Jonathan as in: “And the people said unto Saul, *Shall Jonathan die*, who hath wrought this great salvation in Israel?”[*emphasis mine*]) (1 Samuel 14: 45). This question *Shall Jonathan die*? was asked by the people after Jonathan helped his father to free Israel from the Philistines. What matters here is that the question about the death of Jonathan (*Shall Jonathan die*?) has been intertextually incorporated (with a change of a modal verb) into the novel *Defend the Name*:

*Would Jonathan Die*? That was a question very often heard. It was a good question. His abdication and death were both questionable. And the trouble was otherwise, that the children would while living with him, grow and be like him [*sic*]. The death of Jonathan is unlikely. Because you know he looked always young despite his age. Always young despite his age. That would be quite impossible. But yes. In Babilé, I am sorry, I mean in Jonathan, the impossible was possible [*emphasis added*]. [33, p. 89]

Now the reader can see that “Would Jonathan Die?” (asked by colonized natives of Babilé in above quotation) is a parody of *Shall Jonathan die*…? (asked by people of Israel in the Bible). Here, it should be noted that, in the Bible, Jonathan has been portrayed heroically and by asking *Shall Jonathan die*? the people of Israel were expressing their gratefulness to (and their fear for the death of) their hero Jonathan. However, in *Defend the Name* by Wolde, the character Jonathan has been portrayed villainously as a tyrant and oppressive colonizer and by asking, “Would Jonathan Die?” the colonized natives were expressing their grievances and suffering under the colonial rule led by Jonathan. In short, one may argue that the novelist Wolde’s purpose for intertextually borrowing the name of his character Jonathan from a heroic warrior of the same name in the Bible is satirical and/or ironical, in that our attention would be on differences (rather than on similarities) between the character traits and deeds of the two Jonathans.

To conclude, the intertextual analysis of the references to both the name and death of Jonathan in *Defend the Name* adds depth and complexity to the character. By drawing on the biblical Jonathan, the author establishes a connection that echoes the heroic acts of the biblical figure. However, the parody of the question about Jonathan’s death highlights the contrasting traits and actions of the fictional Jonathan, who is portrayed as tyrannical and oppressive. This intertextual analysis engages readers in examining the stark differences between the two characters and explores the novel’s themes of colonization and power dynamics.

#### 7.2.2. Parody of the “Lord’s Prayer”

The Biblical text of “Lord’s Prayer” has been parodied by Nathnael in his prayer to God to help him wage a successful revolution to overthrow the colonial regime. It was when Nathnael and his friend were discussing the necessity of revolution that Nathnael first remarked: “It is already bred; the time is ripe” so that it is a golden opportunity that he “shall not miss,” and then he began to pray as follows:

“God, praise[d] be thy name! Let the houses overturn, let us overcome the hypocrites, let there come a civil war, a bitter one and let vanish the taste that is sour and what more?” [33, p. 99]

This text of a prayer by Nathnael is a parody because it imitates the tone of the Lord’s Prayer as a pre-text. But, according to Robert Phiddian [39], though parody is an imitative practice, the activity of parody depends on Jacques Derrida’s notion of “writing under erasure” of the “pre-existing text(s) or discourses, so that it can be said that these verbal structures are called to the readers’ minds and then placed under erasure” [39, pp. 13–14, as quoted in 40, p. 15]. By implication, the above-quoted text of a prayer by Nathnael is a parodic “writing under erasure” of some of the verbal structures and tone of the Lord’s Prayer inscribed in Luke 11: 1–13, of which the first few lines include:

Our Father which art in heaven, Hallowed be thy name. Thy kingdom come. Thy will be done, as in heaven, so in earth.

Give us day by day our daily bread.

And forgive us our sins; for we also forgive every one that is indebted to us. And lead us not into temptation; but deliver us from evil. (Luke 11: 2–4)

In other words, by parodying this text of the Biblical prayer, the character Nathnael in *Defend the Name* has combined the spiritual and the secular (i.e., political) subjects. By so doing, Nathnael was mocking the spiritual tone of Christianity, the reason for which could possibly be because Christianity had served the white man Jonathan well in colonizing and thereby subjugating the indigenous black people of Babilé whose queen regnant had been Nathanel’s mother lady Babilé. Here, the instrumentality of the Christian religion for the colonization and enslavement of the people of Babilé might remind the reader of Karl Marx’s calling religion “the opium of the people,” also termed “the opiate of the masses” [41, p. 59]. As a result, from the perspective of the Marxist literary criticism, Nathnael’s parodying of the “Lord’s Prayer” (in *Defend the Name*) might be construed as a subversive (or deconstructive) critique of the Christian religion, that is, as an attempt on Nathnael’s part to ridicule the serious spiritual tone of the “Lord’s Prayer,” which has been misappropriated for colonialist and imperialist interests. Theoretically [40], explains that, as opposed to the “conservative” function of parody, “there is another tradition which celebrates the subversive possibilities of parody as its essential characteristic; parody in this view typically attacks the official word, mocks the pretensions of authoritative discourse, and undermines the seriousness with which subordinates should approach the justifications of their betters” [37, 40].

However, in *Defend the Name*, one of Nathnael’s friends, who was listening to Nathnael’s long discourse on a revolution, eventually became interested just in making impractical discussions on the subject of revolution rather than waging actual revolution: “We need it. We need a revolution. *Revolution*, *revolution*, *revolution I like the word*. *I would be happy to always discuss revolutions*” [*emphasis mine*] [33, p. 100]. As the reader can see from the lines quoted here, this character has thrice repeated the word *revolution*, just because he likes the word as a word devoid of performativity; hence, his declarative statement “I like the word” is a metafictional comment on his liking for the word *revolution* and on his being happy to always engage himself in discussing it theoretically. This self-reflexive or metafictional comment is a narrative strategy which signifies the fictionality of the narrative in question.

The analysis of the parody of the "Lord’s Prayer" in *Defend the Name* holds intertextual significance within the novel. Through this parody, the character Nathnael engages in a subversive critique of Christianity and its complicity in colonization and subjugation. By imitating the structure and tone of the biblical prayer, Nathnael exposes the misuse of spirituality for colonialist and imperialist purposes. This act of parody aligns with a tradition that challenges authoritative discourse and ridicules the pretensions of those in power. Furthermore, the metafictional comment made by one of Nathnael’s friends highlights the narrative’s fictional nature. The intertextual significance of this analysis lies in its exploration of the complex dynamics between religion, power, and resistance within the context of the novel.

### 7.3. References to modern ideological and theoretical texts in *Defend the Name*

As stated in the paratextual introduction to *Defend the Name*, an “appeal to conscience” is one of the novel’s strategies for the anticolonial resistance. Hence, in connection to this theme, lady Babilé speaks of her having read theoretic/ideological books on (cultural) materialism, colonialism and religion. These texts are referred to by Lady Babilé in her question to her old maidservant Tolulu, “Have you read *Materialism* by Kerri, *Colonialism* by Tinksten and *Influence of Religion on Human Lives* by Leon?” [33, p. 21]. From the perspective of intertextuality, lady Babilé’s direct references (and/or verbal allusions) to such theoretical/ideological (or cultural) texts signify the importance of exploring how Wolde’s novel *Defend the Name* is thematically related to each of these texts referred to by lady Babilé as a revolutionary character.

Moreover, in her efforts to raise the consciousness of her people to struggle for their freedom, lady Babilé speaks in support of the emergence of anticolonial critics and the importance of reading and assimilating writings on Marxist theories of socialism and revolution. To this effect, she tells Tolulu, “There have come men whose humorous writings have rocked with me with laughter” and “There have also come men whose writings have revolutionized the whole society” [33, p. 21].

Meanwhile, she remarks on the roles played by literary artists (poets, dramatists and fiction writers) in educating the oppressed (or the wretched of the earth) so that they could better understand the multifaceted ideological damages wrought about by colonialism. Likewise, lady Babilé is heard acknowledging the emergence of educated critics and commentators in the community as well as expressing her gratefulness to them, for they have spoken of Jonathan’s true colours; as she reports it to Tolulu, “They say that Jonathan is one intoxicated with the miasma of power, a blood-sucker, an exploiter, an executor of injustice” [33, p. 21]. On top of her intertextual references attributed to the modern/contemporary ideological/theoretical texts, lady Babilé has written a book of history by “tracing the origins of colonialism to the present day dictatorship” [33, p. 56]. Her new book, as she tells her son, serves to subvert the falsifications and biases of writings by Jonathan and his like.

In conclusion, the references to modern ideological and theoretical texts in *Defend the Name* hold intertextual significance, as they establish thematic connections and highlight the novel’s engagement with these ideas. Lady Babilé’s mention of books on materialism, colonialism, and the influence of religion on human lives signals the importance of exploring these themes within the story. Her endorsement of anticolonial critics, emphasis on Marxist theories, and acknowledgment of the role of literary artists and educated commentators further underscore the novel’s alignment with resistance and the power of critical voices. Lady Babilé’s own book, which challenges the falsifications of the oppressive regime, adds a subversive layer to the intertextual significance, as it exposes biases and offers an alternative perspective. Overall, the analysis of these references deepens the exploration of power dynamics, resistance, and the struggle for freedom in the context of colonialism within Defend the Name.

### 7.4. Cultural and historical discourses as sources of intertextuality in *Defend the Name*

#### 7.4.1. The discourses of the “self-made man” and colonialism

Both discourses of the “self-made man” and going from “rags-to-riches” in life have been intertextually evoked in the characterization of Jonathan in *Defend the Name*. The very first chapter of the novel starts off by describing Jonathan’s impoverished and penurious life:

The almost age-withered Jonathan could no longer survive in bitter poverty. His unkempt hair was always hidden under a battered hat. His rugged face wore an appearance of a vindictive animosity. One could not miss the sight of his clothing with a variety of patchwork. And his sandals, well-worn, were beyond repair. The effect was that of a man who did not possess a penny. [33, p. 1]

Here, the dominant imagery depicts Jonathan’s body as being scathed by age and destitution. The worst of it is the narrator’s remark, in the next paragraph, that Jonathan’s “[s]uffering mounted beyond the endurable,” in that he “reached a point where he lost the sight of food for days on end, and where his shabby clothing exposed his naked body”. However, despite the despairing predicament he is in, Jonathan not only “had always been thinking of possible solutions to problems of this kind” but also “did not stop fighting his way up the steep ladder of life” [33, p. 1]. At this stage the reader is in a position to show how Jonathan’s characterization relies on the interrelated discourses of going from rags-to-riches life and the self-made man.

Based on the description provided, one can argue that Jonathan is planning to transition from a state of poverty or destitution to wealth or success, thus aiming to become a self-made individual. Of course, as a means to the attainment of this, Jonathan has vowed to destroy his sworn enemies—namely, famine and infamy—as reported by the narrator in: “And audacious Jonathan is going to wage war against famine and infamy” [33, p. 8]. Moreover, in the passage we have quoted earlier, there is a sentence in which the narrator describes Jonathan’s facial features: “His rugged face wore an appearance of a vindictive animosity” [33, p. 1]. The term “rugged” (in “His rugged face”) is found to have discursive, intertextual implications. According to the *OED*, one of the meanings of the word “rugged” means a person “determined to succeed in a difficult situation, even if this means using force or upsetting other people” and this, as applied to Jonathan’s facial features, is suggestive of the potential self-made man. Put simply, when the adjective *rugged* is used to describe an individual’s facial features, it suggests a strong-featured physical strength and/or the strength of character. In fact, this meaning of *rugged* is in turn rooted in the social ideal of *rugged individualism*, as described by Tyson (2006) in relation to the American dream:

*Rugged individualism*, which… is a cornerstone of the American dream, is an ideology that romanticizes the individual who strikes out alone in pursuit of a goal not easily achieved, a goal that often involves risk and one that most people would not readily undertake. In the past, such a goal would have been, for example, the rush for gold and silver on the American frontier, an attempt in which many individuals risked losing their lives. Today, such a goal might be the undertaking of a high-risk business, in which one risks losing all one’s money. Although it may sound like an admirable character trait, Marxist thinkers consider rugged individualism an oppressive ideology because it puts self-interest above the needs—and even above the survival—of other people. By keeping the focus on “me” instead of on “us,” rugged individualism works against the well-being of society as a whole and of underprivileged people in particular. Rugged individualism also gives us the illusion that we make our own decisions without being significantly influenced by ideology of any sort when, in fact, we’re all influenced by various ideologies all the time, whether we realize it or not. [41, p. 60]

It was on the basis of this ideology of rugged individualism that the character Jonathan in *Defend the Name* decides to leave for the fictive land of Babilé where he would become a self-made man. As reported by the narrator,

“Today,” he [Jonathan] said, “marks the line of demarcation between success and failure. This departure of mine should be viewed as decisive factor for the fulfilment of my goals, of my needs and desires, of my needs and plans, of my ambitions and aspirations. It serves also as a conclusive evidence that there are reactions to all actions, that the so-called laws of nature could be condemned and violated when they conspire. A new dawn of light has fallen upon me. I believe that richness, happiness, improvement and progress are in my own hands and not in the hands of some blind destiny. I can no longer wait for the amelioration of my present conditions by fate. It has enough fooled me. I now understand that it is no mere wrath of the gods that has made me poor. It is the sole inability of myself to react to it. And with this realization I denounce my citizenship of this Earth. And audacious Jonathan is going to wage vendetta against famine and infamy…” [33, p. 8]

By contrast, in the passage by Tyson (2006) quoted earlier, it is necessary to recall that the discourse of the self-made man (or rugged individualism) is decried by Marxist thinkers because of its being based on ruthless egoism, which proves harmful to the interest and well-being of others. Then what matters here is that this ruthless egoism of the rugged individual is found to characterize the conduct and behavior of Jonathan in *Defend the Name*. This can be illustrated by his contemplation of exterminating anyone who dares to stand against his interests, as in: “Jonathan is unbeatable. Let my enemies be careful before it is too late. If there are any, let them be prepared to leave my regime or to entertain a good deal of punishments. For me this will not at all be a painstaking task. Bullets to the culprits, death to the offensive!” [33, p. 5].

The analysis of the discourses of the "self-made man" and colonialism in *Defend the Name* establishes a connection between Jonathan’s character and broader socio-cultural and historical contexts. Jonathan’s journey from poverty to success aligns with the narrative of the "self-made man," highlighting his determination to overcome adversity and achieve wealth and power. This intertextual reference prompts readers to consider the ideals of rugged individualism and its consequences. The intertextual link between the "self-made man" and colonialism deepens the narrative by emphasizing the similarities in their prioritization of personal gain and domination. Jonathan’s ruthless egoism reflects the oppressive nature of colonialism. By intertwining these discourses, the novel encourages readers to critically examine the consequences of unchecked ambition, egoism, and power pursuit, raising ethical and societal questions. The intertextual significance enriches the exploration of power dynamics, morality, and individual responsibilities in society.

#### 7.4.2. The discourses of the mission(ary) and colonialism

The history of the advent of European Christian missionaries into different African nations has been usually disputed to be economically motivated; thus, the historical discourses on the processes of colonialism are shot through with ideological discourses and debates on the motives and pros and cons of Christianity in Africa. Such historical and ideological issues of the colonial encounter have always been intertextually recreated and reflected in works of African writers and artists. In this respect, an attempt would be made to analyze how the Ethiopian novelist Wolde has intertextually drawn in his novel, *Defend the Name*, on the discourse of the Christian mission in relation to the theme of colonialism.

The plot of Wolde’s novel, as stated before, begins with the flight of an “almost age-withered” white man, Jonathan, to a fictive city of Babilé which, according to Jonathan, is located “high up in the universe between the Heavens and the Earth” [33, p. 4]. Upon landing in this city, Jonathan self-proclaims to be a missionary. However, the beginning of the story (i.e., the exposition part of the plot) seems to be replete with motifs and discourses of the black indigenous people’s initial encounter with the European missionary cum colonizer. In the first place, the fact that the city/land of Babilé is inhabited by black people is evocative of the discourses of the Christian mission and colonialism (and/or imperialism). This, in turn, calls to mind the interrelated ideological discourses and literary themes, such as that the black race is intrinsically inferior to the white race and that the blacks are barbaric (subhuman), heathen, *alien*/*other*, hence the “essentiality” of Christianization and colonization. Secondly, the narrator’s characterization of Jonathan on the very first page of the novel can be interpreted intertextually:

The almost age-withered Jonathan could no longer survive in bitter poverty. His unkempt hair was always hidden under a battered hat. His rugged face wore an appearance of a vindictive animosity. One could not miss the sight of his clothing with a variety of patchwork. And his sandals, well-worn, were beyond repair. The effect was that of a man who did not possess a penny. [33, p. 1]

From the viewpoint of intertextuality, images of Jonathan’s bitter poverty (or his pennilessness), of his ragged/rugged clothing and sandals (and of even his unkempt hair) might evoke the same apostolic images of John the Baptist of the Bible, who has been pictured to be raggedly/ruggedly dressed and lacking even the bare necessities of life (as in: “And the same John had his raiment of camel’s hair, and a leathern girdle about his loins; and his meat was locusts and wild honey” [Mtt. 3: 4]). Luckily, the above argument for the novelist’s reliance on the images (or/and the figure) of John the Baptist and on his apostolic calling for (or/and vocation of) preaching and undertaking “the baptism of repentance for the remission of sins” (Luke 3: 3) can be augmented by Jonathan’s reference to “repentance” in his announcement of his mission to the natives of Babilé [42, p. 9].

The reader can, therefore, argue that, by intertextually drawing the discourse of the abused missionary vocation into the plot of his novel *Defend the Name*, the Ethiopian writer Wolde has been able to satirize Christianity’s instrumentality for colonization (and imperialism). In other words, Jonathan’s intent to depart for a foreign land named Babilé was based on two contradictory expectations of the Christian missionary and colonialism/ imperialism, involving his explicit economic motives, which had been already described by the narrator:

But he warned himself not to tell anybody about his destination. He even dared not venture to speak of his departure. It might have been jealousy on his part or reluctance to accept companions. He was, though relieved a little, beset by yet to come problems. He thought if there were no living things in Babilé he would import just a few loyal servants and establish a government of his own by his own and for his own. But if there were harmless beings, he would preach the Gospel, would teach about the life after death, would bring them salvation and a new way of life. But what if there were noxious creatures? This was what he said, “I shall harm the harmful, I shall fight the devil and destroy all vicious elves. I shall not spare a moment to wipe out mischief from my domain. It shall be a respected domain. I shall root out all injustice, all disharmony, all tyranny, all discordance and all dissatisfying elements. Jonathan is unbeatable. Let my enemies be careful before it is too late. If there are any, let them be prepared to leave my regime or to entertain a good deal of punishments. For me this will not at all be a painstaking task. Bullets to the culprits, death to the offensive!

I am highly disappointed. And these disappointments sprang from my economic situation. I should be quick and active to overcome the financial distress I am confronted with. Alas! I must not look upon my sagacity as a panacea for all my economic ills. Man composes and God disposes. I believe in the Almighty, in Him who shall change my dreams into reality. [33, p. 5]

Then, arriving at lady Babilé’s house in the city, Jonathan announces his mission to be there: “I am the Honorable Reverend Jonathan. I am the rightful Messiah, or if please, the Ambassador and plenipotentiary of God. I have come here because I feel I have a Moral Responsibility. I have come here to patronize you—you who need my patronage, to summon you to *repentance*, you who need *repentance* [*emphasis mine*]” [33, p. 9]. Here, Jonathan has advisedly employed some words which have intertextually discursive meanings and thereby which can invite double (or multiple) interpretations of his intentions. For instance, the English word *ambassador* “is a highly cosmopolitan word” whose ultimate origin is the same as that of *embassy* [43, p. 21]; as a result, the *OED* [44] defines the word *ambassador* as “an official who lives in a foreign country as the senior representative there of his or her own country”. Likewise, the dictionary meaning of the word *plenipotentiary* is “a person who has full powers to take action, make decisions, etc., on behalf of their government, especially in a foreign country” [44]. Thus, the plural meanings of both words seemed to have helped Jonathan to play out his double entendre as missionary-colonialist-imperialist settler.

Further, issues of gender and power relations between the black and the white races are implicated by two other words used by Jonathan; namely, the verb *patronize* and the noun *patronage*. Etymologically, these words are rooted in the Latin *pater*, meaning father, and hence, from the feminist viewpoint, these words signify the inferiority of motherhood (femininity) to fatherhood (masculinity). In the case of *Defend the Name*, this seems to be represented by the feminine (matronymic) country/city name Babilé, and, because this name renders the country as a *motherland* (as opposed to the *fatherland*, Jonathan has chauvinistically criticized the queerness and ugliness of the name, belittled it and made the land/city to be name after him:

“Yes I am satisfied with these achievements and advancements. But I can be more satisfied. Really! I can be fully satisfied if this land gets another name. Its present name does sound queer and ugly. It would be to our advantage if we offer it a respectable name. Hence it shall hereinafter be known as “*The city of Jonathan*.” [33, pp. 18–19]

In general, each of these words used by Jonathan (*ambassador*, *plenipotentiary*, *patronage*, *patronize*, *repentance*) have multiple meanings, connoting his religious, political and economic (colonialist/imperialist) desires, on the one hand, and, on the other, the ideological stereotypes about the inferiority and “sinfulness” of the black race represented by natives of Babilé. (To this end, as paratextually stated in the introduction to the novel, after gaining lady Babilé’s trust under such false pretenses or religious hypocrisies, Jonathan at last turns out to be a godless rascal, megalomaniac and despotic colonizer.) Moreover, before the arrival of Jonathan, the black community of Babilé was a matriarchate, and thus Jonathan’s subsequent disavowal of the matriarchal country/city name Babilé betrays the fact that he comes from a predominantly patriarchal society with an ideology which is harmful to women. On the other hand, Jonathan has changed the rightful native name of the city cum country, which is Babilé, and renamed it as *The City of Jonathan* after/for himself and in his honor, which, seen from the perspective (post)colonial literary critical enterprise, might be construed as an example of the colonial *cultural assimilation*.

In summary, the intertextual analysis of the discourses of the mission(ary) and colonialism in *Defend the Name* holds significant literary and socio-cultural significance. By drawing on the historical context of European Christian missionaries and their role in colonialism, the novel explores the complexities and power dynamics inherent in the colonial encounter. The intertextual references to missionary imagery, such as Jonathan’s portrayal akin to John the Baptist, serve to satirize the instrumentalization of Christianity for colonization and imperialism. This critique highlights the economic motives and contradictions underlying Jonathan’s mission, revealing the exploitative nature of colonial enterprises. Additionally, the renaming of the matriarchal city of Babilé to "The City of Jonathan" symbolizes the erasure of indigenous culture and the imposition of colonial cultural assimilation. Overall, the intertextual analysis of the discourses of the mission(ary) and colonialism enriches the novel’s exploration of power, ideology, and the consequences of cultural domination, inviting readers to critically examine the historical and socio-cultural factors shaping colonial encounters and their lasting effects.

## 8. Conclusion and implications

### 8.1. Conclusion

The intertextual analysis of the novel *Defend the Name* reveals a profound exploration of colonialism and its aftermath through the skillful integration of various intertexts. By engaging with colonial literary tropes, biblical intertexts, references to modern ideological and theoretical texts, as well as cultural and historical discourses, the novel creates a multi-layered narrative that vividly portrays the oppressive nature of colonialism, challenges religious authority, explores power dynamics, resistance, and identity formation, and offers valuable insights into the complexities of colonial encounters. This prompts critical reflection on historical and contemporary power structures and their impact on individuals and societies, fostering a nuanced understanding of the lasting implications of colonialism and encouraging a more empathetic approach to the complexities of colonial dynamics.

### 8.2. Implications

The analysis encourages critical engagement with postcolonial issues, emphasizing historical and cultural contexts. It prompts reflection on colonialism’s consequences, challenges religious authority, and explores power dynamics. By incorporating contemporary ideas, it fosters nuanced perspectives on resistance and identity. Overall, *Defend the Name* deepens understanding of colonialism’s impact and encourages critical examination of power and identity in historical and modern contexts, fostering empathy and nuanced views on colonial encounters.
